# Estimating the Trunk Transverse Surface Area to Assess Swimmer’s Drag Force Based on their Competitive Level

**DOI:** 10.2478/v10078-012-0019-3

**Published:** 2012-05-30

**Authors:** Tiago M Barbosa, Jorge E Morais, Mário J Costa, Jean E Mejias, Daniel A Marinho, António J Silva

**Affiliations:** 1 Department of Sports Sciences, Polytechnic Institute of Bragança, Bragança, Portugal.; 2 Department of Sports Sciences, University of Beira Interior, Covilhã, Portugal.; 3 Department of Sports Sciences, University of Trás-os-Montes and Alto Douro, Vila Real, Portugal.; 4 Research Centre in Sport, Health and Human Development, Vila Real, Portugal.

**Keywords:** validation, frontal surface area, drag, gender, expertise

## Abstract

The aim of this study was to compute and validate trunk transverse surface area (TTSA*)* estimation equations to be used assessing the swimmer’s drag force according to competitive level by gender. One group of 130 swimmers (54 females and 76 males) was used to compute the TTSA estimation equations and another group of 132 swimmers (56 females and 76 males) were used for its validations. Swimmers were photographed in the transverse plane from above, on land, in the upright and hydrodynamic position. The TTSA was measured from the swimmer’s photo with specific software. It was also measured the height, body mass, biacromial diameter, chest sagital diameter (CSD) and the chest perimeter (CP). With the first group of swimmers it was computed the TTSA estimation equations based on stepwise multiple regression models from the selected anthropometrical variables. The TTSA prediction equations were significant and with a prediction level qualitatively considered as moderate. All equations included only the CP and the CSD in the final models. In all prediction models there were no significant differences between assessed and estimated mean TTSA. Coefficients of determination for the linear regression models between assessed and estimated TTSA were moderate and significant. More than 80% of the plots were within the 95% interval confidence for the Bland-Altman analysis in both genders. So, TTSA estimation equations that are easy to be computed by coached and researchers were developed. All equations accomplished the validation criteria adopted.

## Introduction

Aquatic locomotion is for human beings quite challenging since they attempt to displace in a different environment they are used to. Comparing human locomotion, in aquatic environment, with fishes and aquatic mammals, the first present a lower efficiency because they have a higher drag force and a lower propulsive ability (Ungerechts, 1983; Ohlberger et al., 2006). That is the reason why so much effort is done by researchers to understand the role of drag force in several human aquatic locomotion techniques, as it is the case of the competitive swimming strokes.

Drag force is dependent from several hydrodynamic and morphometric variables including velocity, shape, size, surface area (Kjendlie and Stallman, 2008):

(1) $$D=\frac{1}{2}\cdot \rho \cdot v^{2}\cdot S\cdot c_{d}$$

Where *D* is the drag force in [N], ρ is the density of the water in [kg·m^−3^], *v* is the swimming velocity in [m·s^−1^], *S* is the projected frontal surface area of the swimmers in [cm^2^] and *C_d_* is the drag coefficient [dimensionless] (changing owning to shape, orientation and Reynolds number).

In this sense, to assess drag force it is needed to collect some selected morphometric variables, as the projected frontal surface area. A couple of techniques to assess drag force insert that specific variables, e.g., computer fluid dynamics (Silva et al., 2008; Marinho et al., 2010a) and velocity perturbation method (Kolmogorov and Duplischeva, 1992; Kolmogorov et al., 2000). When performing a competitive swimming stroke, the subject is in the horizontal position. Therefore, the projected frontal surface area corresponds mostly, but not exactly, to the trunk transverse surface area (TTSA) (Nicolas et al., 2007; Nicolas and Bideau, 2009; Zamparo et al., 2009).

For research, training control and evaluation purposes *TTSA* can be: (i) measured directly with planimeter techniques, on screen measure area software of digital images, or body scans (e.g., Nicolas et al., 2007; Caspersen et al., 2010); (ii) estimated based on some selected morphometric variables (e.g. Clarys, 1979; Barbosa et al., 2010). Although the higher accuracy of measured *TTSA* the procedures are very time consuming, complex and expensive. That is the reason why, in some specific cases, the *TTSA* estimation procedure is the most suitable one.

To the best of our knowledge there is reported in the literature a couple of procedure to estimate the *TTSA* based on selected anthropometrical variables. the subject’s body mass and body height. In one of these procedures, the estimation was developed for young active males (i.e., physical education students) and male world-ranked swimmers (i.e., Olympic swimmers) (Clarys, 1979):

(2) TTSA=6.9256⋅BM+3.5043⋅BH−377.156

Where *TTSA* is the trunk transverse surface area in [cm^2^], *BM* is the body mass in [kg] and *H* is the body height in [cm]. In the other procedure, it were developed and validated *TTSA* estimation equations, respectively, for both males (R^2^ = 0.32; R_a_^2^ = 0.30; s = 158.93; p < 0.01) (Morais et al. 2011):

(3) TTSA=6.662⋅CP+17.019⋅CSD−210.708

And female swimmers with no distinction of their competitive level (R^2^ = 0.34; R_a_^2^ = 0.31; p < 0.01) (Morais et al., 2011):

(4) TTSA=7.002⋅CP+15.382⋅CSD−255.70

Where *TTSA* is the trunk transverse surface area in [cm^2^], *CP* is the chest perimeter in [cm] and *CSD* is the chest sagital diameter in [cm]. So, it seems to exist a chance to develop *TTSA* estimation equation according to the swimmers competitive level (expert versus non-expert swimmers) according to his/her gender. So, the study of Morais et al. (2011) aimed to estimate TTSA only according to gender. It is known that are morphometric differences according to the swimmer’s skill level (competitive vs non-competitive swimmers for the same gender). However, it seems there are not in the literature such TTSA estimation equations. For some practitioners and researcher equations even more accurate, according to the subjects characteristics can be very useful. For instance, to be able to estimate TTSA not only based on gender but on the swimmer’s skill level as well.

The aim of this study was to compute and validate *TTSA* estimation equations to be used assessing the swimmer’s drag force according to gender and competitive level. It was hypothesized that it is possible to compute accurate and valid equations to estimate *TTSA* for both male and female swimmers based on their competitive level (expert and non-expert swimmers).

## Material and Methods

### Sample

**Figure 1:**
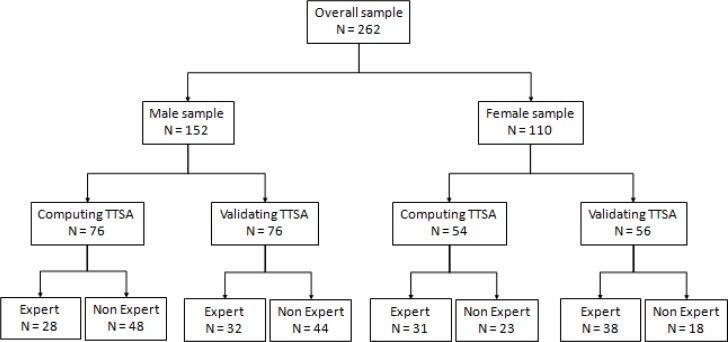
The split of overall sample to compute and validate the trunk transverse surface area (TTSA)

Total sample was composed of 262 subjects (152 males and 110 females). Swimmers chronological ages ranged between 10 and 32 years old for male subjects and 09 and 27 years old for female ones. Total sample was divided in several cohort groups based on gender and competitive level. One group of 130 swimmers (54 females and 76 males) was used to compute the TTSA estimation equations and another group of 132 swimmers (56 females and 76 males) were used for its validations. Overall sample was split in 60 male and 69 female expert swimmers plus 92 male and 41 female non-expert swimmers. It was considered as expert swimmers those participating on regular basis in national and international level competitions. It was defined as non-expert swimmers the ones participating on regular basis in swimming classes and/or in regional level competitions. Figure 1 presents the split of the overall sample.

All procedures were in accordance to the Declaration of Helsinki in respect to Human research. The Institutional Review Board of the Polytechnic Institute of Bragança approved the study design. Subjects (or when appropriate their legal tutors) were informed of the potential experimental risks and signed an informed consent document prior to data collection.

### Data collection

**Figure 2:**
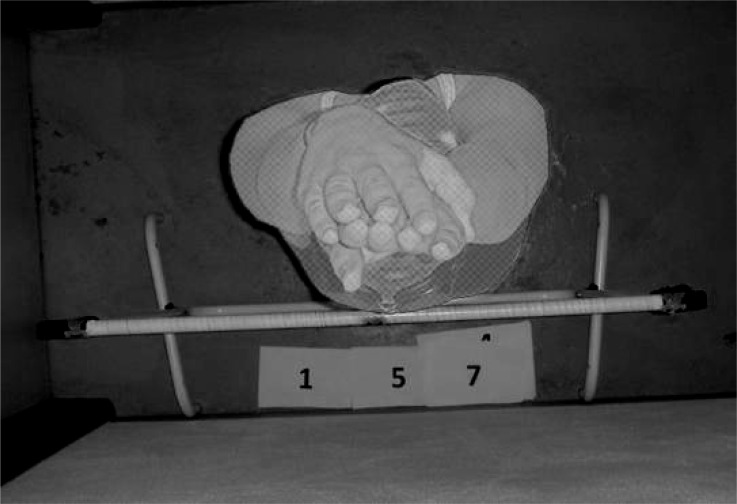
Manual digitization of the trunk transverse surface area (TTSA)

For the *TTSA* measurement, subjects were photographed with a digital camera (DSC-T7, Sony, Tokyo, Japan) in the transverse plane from above (Caspersen et al., 2010; Morais et al., 2011). Subjects were on land, in the upright and hydrodynamic position. This position is characterized by the arms being fully extended above the head, one hand above the other, fingers also extended close together and head in neutral position. Subjects wear a regular textile swim body suit, a cap and goggles. Besides the subjects, on the camera shooting field was a calibration frame with 0.945 [m] length at the height of the xiphoid process (Figure 2). *TTSA* was measured from the subject’s digital photo with specific software (Udruler, AVPSoft, USA). Procedures included: (i) scale calibration; (ii) manual digitization of the transverse trunk perimeter; (iii) output and recording of the *TTSA* value.

It was also measured the following selected anthropometrical variables: (i) body mass; (ii) height; (iii) biacromial diameter; (iv) chest sagital diameter and; (v) chest perimeter. Most of these variables are reported on regular basis in competitive swimming anthropometrical reports and research papers (e.g., Mazza et al., 1994). All measurements were carried-out once again wearing a regular textile swim body suit, a cap and goggles. Body mass (BM) was measured in the upright position with a digital scale (SECA, 884, Hamburg, Germany). Height (H) was measured in the anthropometrical position from vertex to the floor with a digital stadiometer (SECA, 242, Hamburg, Germany). Biacromial diameter (BCD) is considered as the distance or breadth between the two acromion processes. Chest sagital diameter (CSD) is considered as the distance or breadths between the back and the highest point of the chest (i.e. antero-posterior) at the level of the xiphoid process. Both diameters were measured once again with a specific sliding calliper (Campbell, 20, RossCraft, Canada), being the subjects in the anthropometrical position (both foot on the ground, in an orthostatic position, both arms in lateral abduction at a 90° angle with the trunk) and inspiratory apneia. Chest perimeter (CP), defined as the perimeter of the trunk at the level of the xiphoid process, was measured with a flexible anthropometrical tape (RossCraft, Canada). An expert evaluator performed all anthropometrical evaluations. Three measures of each anthropometrical variable were conducted. For further analysis the mean value of all three trials was considered.

### Statistical procedures

The normality and homocedasticity assumptions were checked respectively with the Kolmogorov-Smirnov and the Levene tests. Descriptive statistics (mean, one standard deviation, minimum, maximum and coefficient of variation) of all measured variables were calculated.

For a given sub-sample group (i.e., non-expert sub-sample and expert sub-sample groups in each gender) forward step-by-step multiple regression models were used to compute the *TTSA* estimation models. For the *TTSA* estimation in the overall sample group in each gender based on the competitive level (i.e., males and females sample groups) this one was inserted as a dummy variable (0 = non-expert swimmer; 1 = expert swimmer). *TTSA* was considered as endogenous variable and remaining anthropometrical variables (i.e., body mass, height, *BCD*, *CSD* and *CP*) as exogenous variables. The variables entered the equation if F ≥ 4.0 and removed if F ≤ 3.96 as suggested elsewhere (Barbosa et al. 2008). All assumptions to perform the selected multiple regression models were taken into account. It was considered for further analysis the computed equation, the coefficient of determination (R^2^), the adjusted coefficient of determination (R_a_^2^), the error of estimation (s) and the probability of rejecting the null hypothesis (p ≤ 0.05). In each exogenous variables included in the final model, the t-value and the p-value were considered as well.

Validation was made in a second sub-sample group (Morais et al., 2011): (i) comparing mean data; (ii) computing simple linear regression models and; (iii) computing Bland Altman plots. Comparison between the mean *TTSA* assessed and the *TTSA* estimated, according to the equations previously developed, was made using paired Student’s t-test. It was defined as validation criteria that there was not significant differences between pair wise data (p > 0.05). Simple linear regression model between both assessed and estimated *TTSA* was computed. As a rule of thumb, for qualitative interpretation, effect size analysis and validation criteria, it was defined that the relationship was: (i) very weak if R^2^ < 0.04; weak if 0.04 ≤ R^2^ < 0.16; moderate if 0.16 ≤ R^2^ < 0.49; high if 0.49 ≤ R^2^ < 0.81 and; very high if 0.81 ≤ R^2^ < 1.0. In addition, it was computed the error of estimation (s) and the confidence interval for 95% of the adjustment line in the scatter gram. Bland Altman analysis (Bland and Altman, 1986) included the plot of the mean value of *TTSA* assessed and estimated versus the delta value (i.e., difference) between *TTSA* assessed and estimated. It was adopted as limits of agreement a bias of ± 1.96 standard deviation of the difference (average difference ± 1.96 standard deviation of the difference). For qualitative assessment it was considered that *TTSA* estimated was valid and appropriate if at least 80% of the plots were within the ± 1.96 standard deviation of the difference.

## Results

### Morphometric characteristics

Tables 1 and 2 present the descriptive statistics for all selected anthropometrical variables in each competitive level sub-sample group. Data dispersion can be considered as ranging from weak (i.e., CV ≤ 15 %; e.g., *H* or *CP*) to moderate (i.e., 15 % < CV ≤ 30 %; e.g., *BM* or *TTSA*) within each sub-sample group. It can be verified that all mean values are higher in male than in female for the expert sub-sample groups, but there were no significant differences based on gender for the non-expert sub-sample groups.

Comparing descriptive statistics according to competitive level, it seems that mean values are very close but smoothly higher in the non-expert level sub-sample groups. On the other hand, the CV is higher for the majority of the variables in the expert sub-sample cohorts.

### Computation of trunk transverse surface area prediction models

For male gender, expert sub-sample group, the final model (F_2,27_ = 6.078; p = 0.01) included the *CP* (t = 2.307; p = 0.03) and the *CSD* (t = 1.858; p = 0.08) in order to predict the *TTSA*. The equation was (R^2^ = 0.33; R_a_^2^ = 0.27; s = 165.41; p < 0.01):

(5) TTSA=10.505⋅CP+19.216⋅CSD−575.496

For male gender, non-expert sub-sample group, the final model (F_2,47_ = 20.509; p < 0.001) included in the final models the *CP* (t = 1.050; p = 0.30) and the *CSD* (t = 1.606; p = 0.11). The equation was (R^2^=0.48; R_a_^2^ = 0.45; s = 136.89; p < 0.01):

(6) TTSA=5.030⋅CP+30.453⋅CSD−371.404

For overall male gender group, including the competitive level as dummy variable (0 = non-expert; 1 = expert), the final model (F_3,75_ = 17.001; p < 0.001) included the *CP* (t = 3.253; p < 0.01) and the *CSD* (t = 2.443; p = 0.02) in order to predict the *TTSA*. The equation was (R^2^ = 0.42; R_a_^2^ = 0.39; s = 146.39; p < 0.01):

(7) TTSA=8.413⋅CP+19.984⋅CSD+19.854⋅competitive−414.695

For female gender, expert sub-sample group, the *TTSA* prediction model (F_2,30_ = 5.931; p < 0.01) included the *CP* (t = 2.671; p = 0.01) and the *CSD* (t = 2.063; p = 0.05). The estimation equation was (R^2^ = 0.28; R_a_^2^ = 0.24; s = 147.015; p < 0.01):

(8) TTSA=10.875⋅CP+16.498⋅CSD−504.705

For female gender, non-expert sub-sample group, the final model (F_2,20_ = 3.914; p = 0.04) included the *CP* (t = 2.294; p = 0.03) and the *CSD* (t = 1.145; p = 0.05) in order to predict the *TTSA*. The *TTSA* estimation equation was (R^2^=0.28; R_a_^2^ = 0.21; s = 115.199; p = 0.04):

(9) TTSA=14.836⋅CP+26.825⋅CSD−33.149

For overall female gender group, including competitive level as dummy variable (0 = non-expert; 1 = expert), the *TTSA* estimation model (F_3,52_ = 5.692; p < 0.001) included the *CP* (t = 2.950; p < 0.001), the *CSD* (t = 1.682; p = 0.01) and the competitive level (t = 2.350; p = 0.02) The final equation was (R^2^ = 0.25; R_a_^2^ = 0.21; s = 136.922; p < 0.001):

(10) TTSA=8.457⋅CP+11.614⋅CSD+99.7⋅competitive−322.464

### Validation of trunk transverse surface area prediction models

**Figure 3:**
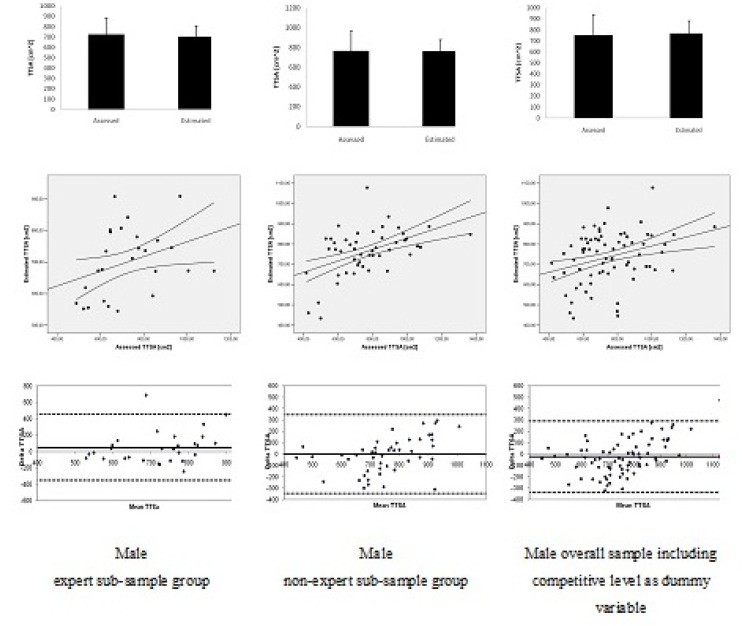
Comparison of mean data, scatter gram and Bland Altman plots between assessed and estimated trunk transverse surface areas (TTSA) for male sub-sample and overall sample groups

**Figure 4:**
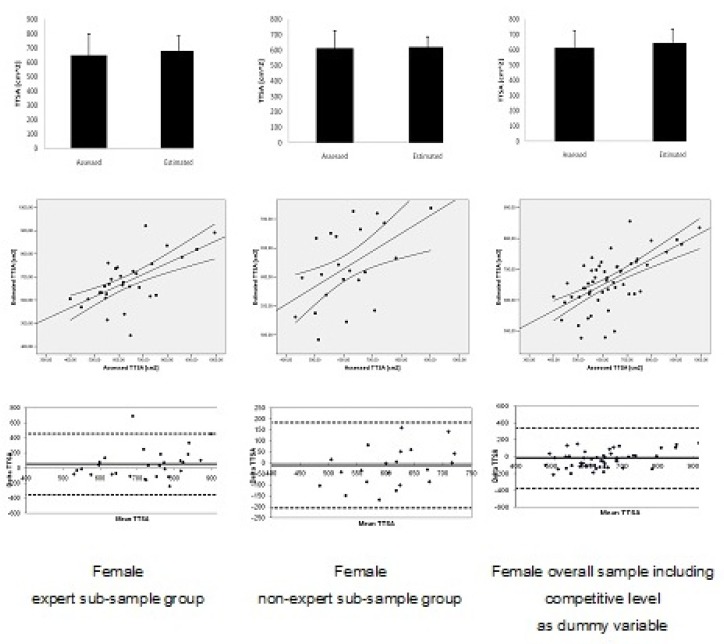
Comparison of mean data, scatter gram and Bland Altman plots between assessed and estimated trunk transverse surface areas (TTSA) for female sub-sample and overall sample groups

Figures 3 and 4 present the validation procedures including the mean data comparison, scatter gram and Bland Altman plots between assessed and estimated *TTSA* based on equations equations 5 to 7 and 8 to 10, for the male and female sub-sample groups, respectively. For all sub-sample groups, in both genders and for polling data in each gender, mean data was non-significant (p > 0.05) comparing assessed and estimated *TTSA*.

Analyzing the scatter grams, all simple linear regression models between assessed and estimated *TTSA* were significant and ranging from moderate to high relationships for the sub-sample groups and the overall sample groups in each gender. For males, relationships ranged between R^2^ = 0.23 (s = 102.41; p = 0.01) and R^2^ = 0.59 (s = 74.44; p < 0.001). For females, relationships ranged between R^2^ = 0.32 (s = 55.73; p = 0.01) and R^2^ = 0.38 (s = 67.28; p < 0.001).

For the Bland Altman plots, all sub-sample groups accomplished the criteria of at least 80% of the plots being within the ± 1.96 SD. Indeed, for the six assessed conditions, only in two of them one single plot was beyond the 95% of agreement limits in the male and female expert sub-sample groups, respectively.

## Discussion

The aim of this study was to compute and validate *TTSA* estimation equations to be used assessing the swimmer’s drag force according to gender and competitive level. All equations computed estimate the *TTSA* based on the *CP* and *CSD* and are valid to such purpose in each gender according to the competitive level.

### Morphometric characteristics

The head, trunk and limb’s actions induce changes on the swimmer’s surface area in the direction of the motion within the stroke cycle. For instance, some previous research reported that lateral body movements and/or ondulatory ones might increase TTSA during fin swimming (Nicolas and Bideau, 2009). The TTSA represents the cross sectional area in the hydrodynamic position and not the projected frontal area. During swimming the body is less streamlined and presents a higher frontal area to the fluid then when in the hydrodynamic position (Zamparo et al., 2009). In spite of not representing the projected frontal area while swimming, the TTSA estimation equations are a less complex and time consuming procedures that might provide useful information for coaches and researchers in order to assess the drag force.

Swimmers morphometric characterization aims to verify to which extend subjects used to estimate *TTSA* and for its validation are representative of remaining ones according to data reported in previous literature. Regarding swimmers dimensions and surface areas assessed, most mean values were higher in male than in female subjects as reported consistently in recent literature (Mazza et al., 1994; Strzała et al., 2005; 2007; Nicolas et al. 2007; Nicolas and Bideau, 2009; Knechtke et al., 2010; Caspersen et al., 2010).

Within each gender, mean values are smoothly higher in the non-expert level sub-sample groups. On other hand, this cohort groups present a lower data dispersion. Expert level groups seem to be more homogeneous than non-expert ones. Non-expert level groups included subjects with several backgrounds, as regular swim classes students, sport and physical education students or competitive swimmers with lower physical fitness shape, competitive level and low training loads. On the other hand, expert level groups included swimmers with somewhat high standard and enrolled on daily basis (twice a day) to very high training loads. Indeed, male and female swimmers are becoming more “androgynous” as differences among them seem to be less obvious nowadays (Barbosa et al., 2006).

So, morphometric characteristics from expert male and female swimmers seem to be more homogeneous, similar to each other. In this sense, subjects selected for this research are very similar to the ones reported in the recent literature.

### Computation of trunk transverse surface area prediction models

The six equation models computed included the *CP* and the *CSD*. The equations were significant and with a prediction level qualitatively considered as moderate. This means that some other latent variables, not inserted in the model, might increase the *TTSA* estimation level. However, the anthropometrical variables selected are easy to collect by coaches and researchers since the apparatus used are less expensive and the data acquisition procedures are quite simple and quick to be performed.

Equations 5 to 10 have a coefficient of determination lower than the equation proposed in by Clarys (1979) and similar or slightly higher than the ones suggested by Morais et al. (2011) to estimate *TTSA*. Regarding the comparison with Clarys (1979) equation, some issues must be addressed: (i) equations 5 to 10 were computed for a broad range of ages and not for a strict age-frame, such as only children or young adults or middle-age adults or elderly; (ii) morphometric characteristics of sub-sample groups are heterogeneous; (iii) from a geometric point of view, perimeters and distances or breadth are the determining variables to compute areas; (iv) to the best of our knowledge the only equation reported in literature until yet was not validated to be used by both male and female genders, no matter their competitive level or chronological age. Regarding the Morais et al. (2011) estimations, the equations presented in this paper are similar or slightly higher because cohort groups are more homogeneous for these last ones.

### Validation of trunk transverse surface area prediction models

Validation for equations 5 to 10 was done using three data analysis techniques: (i) comparing mean data; (ii) computing coefficient of determination and; (iii) computing Bland Altman plots. According to the literature concerning to data analysis, all of these procedures have some strengths and weakness (Bland Altman 1986; Lee et al., 1989; Hopkins 2004; Westgard, 2008). In this sense it was decided to use all the three since they are adopted in most apparatus and/or technique validations.

Validations were carried-out with sub-sample groups with similar profiles (i.e., range of ages, competitive level and morphometric characteristics) of the ones used to compute *TTSA*. It is defined as validation criteria that: (i) there is no significant differences between mean data assessed with gold standard and estimated with the new apparatus and/or technique; (ii) coefficients of determination between both conditions are significant and at least moderate (i.e. R^2^ ≥ 0.16) and; (iii) more than 80% of the Bland Altman plots are within the ± 1.96 SD (i.e., approximately 95% confidence interval agreement limits). In all six *TTSA* equation computed, the validation criteria adopted for the three procedures were accomplished. Mean data between pair wise data is very similar (i.e. non-significant differences) and for the six conditions only one plot in the male expert sub-sample group was beyond the agreement limits. The coefficient of determination criteria was also accomplished. In six coefficients all were moderate or high. Moderate-high coefficients of determination means that some data bias might exist between assessed and estimated measures as happens on regular basis in this kind of procedures.

It can be considered as main limitations of this research: (i) *TTSA* computed are only appropriate for subjects from children (i.e. approximately 6 years-old) to young adult (i.e., approximately 30 years-old) of both genders and not being validate for remaining ages (e.g., toddlers, middle-age swimmers or elderly); (ii) adding or forcing extra anthropometrical variables to enter in the final model might increase the *TTSA* estimation level, but data collection will become more time consuming or expensive; (iii) all models presents a moderate prediction level, so for some specific research designs an assessment instead of an *TTSA* estimation will decrease data bias.

As a conclusion: (i) all morphometric data assessed are within the range of values reported on regular basis for expert and non-expert swimmers of both genders in recent literature; (ii) *TTSA* estimation models computed were significant and with moderate coefficients of determination; (iii) all the validation criteria (mean data comparison, simple linear scatter plots and Bland Altman plots between estimated and assessed *TTSA*) were accomplished. In this sense, it can be stated that the prediction models developed can be used with validity to estimate *TTSA* for both male and female swimmers according to their competitive level.

## Figures and Tables

**Table 1:** Anthropometrical characterization of male (M) and female (F) expert sub-sample groups for the body mass (BM), height (H), biacromial diameter (BCD), chest sagital diameter (CSD), chest perimeter (CP) and measured trunk transverse surface area (TTSA)

	BM [kg]		H [cm]		BCD [cm]		CSD [cm]		CP [cm]		TTSA [cm^2^]	

	M	F	M	F	M	F	M	F	M	F	M	F
Mean	54.83	46.96	164.52	155.88	37.46	34.61	22.44	21.40	81.63	74.83	715.57	642.93
1 SD	11.78	9.71	11.73	9.61	6.34	5.07	3.72	3.24	7.49	7.26	175.51	153.65
Minimum	32.00	27.80	141.00	133.00	19.90	24.20	11.50	15.50	64.00	64.00	417.46	327.21
Maximum	86.00	72.20	188.40	178.00	50.50	44.00	31.00	28.10	100.00	92.00	1371.00	1125.20
CV	21.48	20.68	7.12	6.16	16.92	14.65	16.57	15.14	9.17	9.70	24.52	23.90

P value (M vs F)	<0.001		<0.001		= 0.01		= 0.01		< 0.001		< 0.001	

**Table 2:** Anthropometrical characterization of male (M) and female (F) non-expert sub-sample groups for the body mass (BM), height (H), biacromial diameter (BCD), chest sagital diameter (CSD), chest perimeter (CP) and measured trunk transverse surface area (TTSA)

	BM [kg]		H [cm]		BCD [cm]		CSD [cm]		CP [cm]		TTSA [cm^2^]	

	M	F	M	F	M	F	M	F	M	F	M	F
Mean	69.07	55.43	172.50	160.24	34.12	30.50	22.43	21.88	90.23	83.85	768.48	618.38
1 SD	14.38	8.26	11.38	8.33	3.53	2.99	2.47	1.99	8.81	7.21	188.34	126.71
Minimum	28.00	35.60	134.00	137.00	23.80	25.40	15.40	18.60	61.50	69.00	373.59	355.48
Maximum	108.60	72.20	189.00	172.00	40.20	35.40	30.10	25.60	112.00	97.00	1366.66	959.20
CV	20.81	14.90	6.59	5.19	10.34	17.01	11.01	9.10	9.76	8.60	24.50	20.49

P value (M vs F)	= 0.23		= 0.12		= 0.39		= 0.41		= 0.46		= 0.26	
